# Anti-Inflammatory Effects of *Campomanesia xanthocarpa* Seed Extract Obtained from Supercritical CO_2_

**DOI:** 10.1155/2021/6670544

**Published:** 2021-02-26

**Authors:** Fernanda Petry, Bruna B. Dall'Orsoleta, Mikaela Scatolin, Leticia V. Morgan, Bianca O. Alves, Gabriela S. Anzollin, Gabriela A. L. Zilli, Jaqueline Scapinello, Leticia J. Danielli, Krissie D. Soares, Miriam Apel, J. Vladimir Oliveira, Jacir Dal Magro, Liz G. Müller

**Affiliations:** ^1^Graduate Program in Environmental Sciences, Community University of Chapecó Region (Unochapecó), Chapecó 89809-900, Brazil; ^2^Health Sciences Area, Community University of Chapecó Region (Unochapecó), Chapecó 89809-900, Brazil; ^3^Area of Exact and Environmental Sciences, Community University of Chapecó Region (Unochapecó), Chapecó 9809-900, Brazil; ^4^Graduate Program in Pharmaceutical Sciences, Federal University of Rio Grande do Sul, Porto Alegre 90040-060, Brazil; ^5^Department of Chemical and Food Engineering, Federal University of Santa Catarina, Florianópolis 88040-900, Brazil

## Abstract

*Campomanesia xanthocarpa* is a plant species traditionally used in the treatment of diabetes, fever, hypercholesterolemia, obesity, and urinary tract diseases. The anti-inflammatory effects of *C. xanthocarpa* leaves in mice were already known. Nevertheless, studies on the anti-inflammatory activity of its seeds are still lacking. The aim of this study was to investigate the anti-inflammatory activity and acute toxicity of *C. xanthocarpa* seed extract, obtained from supercritical CO_2_ extraction (SCCO_2_) at 40°C and 250 bar, in mice. GC/MS analysis revealed that *β*-caryophyllene is the major compound present in the *C. xanthocarpa* SCCO_2_ extract. The extract (60 mg/kg, p.o.) significantly reduced the nociceptive behavior in the second phase of the formalin test and prevented the paw oedema induced by carrageenan up to 6 h after carrageenan injection. The extract (0.1–1 *μ*g/mL) inhibited neutrophils migration induced by LPS from *E. coli in vitro*. This antichemostatic effect was comparable to the effect of indomethacin. Acute administration (2000 mg/kg, p.o.) of *C. xanthocarpa* SCCO_2_ extract caused no mice mortality, demonstrating that the extract is devoid of acute toxicity. These data suggest that *C. xanthocarpa* seeds present anti-inflammatory activity and represent a source of anti-inflammatory compounds.

## 1. Introduction

Inflammation is a reaction to infection, irritation, or tissue damage. Some signs of inflammation can be clinically characterized, such as skin redness, heat, swelling, and pain. The human body initiates an inflammatory reaction through a nonspecific immune response that protects the organism from injury and initiates specific immunity responses [1].

The World Health Organization (WHO) estimates that inflammatory diseases are the third largest cause of death in the world, accounting for 3.46 million deaths per year [2, 3]. Consistent with these data, the therapeutic class of nonsteroidal anti-inflammatory drugs (NSAIDs) is widely used worldwide [4]. Around 30 million people in the world use NSAIDs daily, regardless of their adverse events, such as the high risk of gastrointestinal mucosal damage, ulcers and erosions, kidney damage, increased blood pressure, and heart diseases [4]. It is also known that 34–46% of NSAID users present gastrointestinal lesions with a significant risk of perforation or severe bleeding [5].

Another widely used class of anti-inflammatory drugs is steroids, due to their diversity of indications and immunosuppressive effects. These drugs act on both the early and late manifestations of inflammation and are very effective against inflammatory reactions. However, they are associated with several serious adverse effects that modify the body's general metabolism and may be related to the development of Cushing's syndrome after prolonged use [6].

The abundant risks associated with steroid drugs and NSAIDs for the treatment of inflammation suggest the use of herbal medicines as alternative remedies [7]. The technology evolution in the field of phytotherapeutics has allowed a more efficient quality control of drugs based on the identification, determination, and quantification of chemical compounds, which, in turn, makes the manipulation of new herbal products safe, effective, affordable, and with good quality [8].

The plant species *Campomanesia xanthocarpa* Berg. (Myrtaceae) is a tree native to Southern Brazil, popularly known as “guavirova.” This species and other species belonging to the genus *Campomanesia* are popularly used in Brazil for the treatment of diabetes, fever, hypercholesterolemia, obesity, and urinary diseases [9–11]. The antidiarrheal [12], antiulcerogenic [13], antiplatelet [14], hypocholesterolemic [15], antiproliferative, antioxidant, and trypanocidal [16] activities of *C. xanthocarpa* have already been demonstrated. Interestingly, the anti-inflammatory effects of *C. xanthocarpa* leaves in mice were shown in [17], but there are no studies about the effects of *C. xanthocarpa* seeds on the inflammatory process.

Considering the need for the development of new molecular prototypes with anti-inflammatory action, we investigated, for the first time, the effects of *C. xanthocarpa* seed extract obtained by supercritical CO_2_ in animal models of inflammation as well as its acute toxicity.

## 2. Materials and Methods

### 2.1. Plant Extract Material

*C. xanthocarpa* fruits were collected in Quilombo, Santa Catarina, Brazil, in 2017 (26°47′23.6″ S, 52°45′42.41″ W). A voucher specimen was deposited at the Unochapecó Herbarium (SC, Brazil, access #3153). The fruits were kept at 8°C, in the dark, under nitrogen atmosphere. Seeds were separated from the fruits prior to the extraction.

### 2.2. Extraction, Identification, and Quantification of Chemical Compounds

The supercritical CO_2_ (SCCO_2_) extraction was previously carried out by Capelleto et al. [18]. Herein, we tested the biological activity of the previously obtained extract. The extraction was performed in a laboratory extractor. In brief, around 30 ± 0.05 g of seeds (dried in an oven at 40°C to constant weight and then comminuted in a blender −0.14 mm average particle size) were charged into the extraction vessel. The SCCO_2_ flowed at 2 mL/min through the extraction vessel, and the extraction was carried out at 40°C and 250 bar (solvent density of 0.879 kg m^−3^) for 150 min [18, 19].

The identification and quantification of the extract chemical compounds were previously performed by Capelleto et al. [18] by gas chromatography (Agilent GC/MS (7890B)) coupled to a quadripolar mass spectrometer (5977A) (Agilent Technologies, Palo Alto, CA, USA). The GC analysis was performed using Agilent 19091S capillary column (30 m × 250 *μ*m × 0.25 *μ*m). The temperature of the injector and detector was set at 250°C; the oven temperature was programmed from 60°C (8 min) to 180°C (4°C/min), 180–230°C (20°C/min), and then 230°C (for 20 min). Helium was used as the carrier gas at a flow rate of 1.2 mL/min. The chemical components present in the extract were identified in comparison with the equipment library (Agilent P/N G1033A). The relative amounts of each individual component were calculated using their respective peak areas in the chromatogram [18, 19].

### 2.3. Animals

Male and female Swiss mice (25–35 g) from the Unochapecó Bioterium and male Wistar rats (220–250 g) from Centro de Reprodução e Experimentação de Animais de Laboratório (CREAL), Federal University of Rio Grande do Sul (UFRGS), were used. The female mice were used in the toxicity study, and male mice/rats were employed in the other assays.

The animals were kept in acrylic cages, with food and water provided *ad libitum*, in an air-conditioned room (22–24°C) with 12 : 12 h light/dark cycle and controlled air humidity (40–60%). Mice were fasted for 2 hours before oral administrations. The experimental protocols were approved by Animal Care Local Ethical Committee (CEUA-Unochapecó #002–17; CEUA-UFRGS #37366) and performed in accordance with Brazilian law (Lei n. 11.794, de 8 de outubro de 2008) and European Communities Council Directive of 24 November 1986 (86/609/EEC). The solubilization of the extract was carried out in vehicle (0.9% NaCl + 1% Tween 80) and ultrasound. The extracts' doses used were 30, 60, and 120 mg/kg, set after pilot experiments performed in our laboratory and based on literature data [20].

### 2.4. Formalin Test

The formalin test allows the evaluation of two processes: the direct stimulation of the nociceptive fibers at the first moment and the inflammatory reaction (characterized by the release of inflammatory mediators) at the second moment [21, 22]. The test was carried out according to Santos and Calixto (1997) [23]. In brief, the inflammation was induced by intraplantar (i.pl.) administration of 1% formalin (20 *μ*L) in the dorsal region of the right hind paw of the mice. The animals received an oral administration (gavage) of *C. xanthocarpa* extract 1 hour before the exposure to formalin. Mice were observed immediately after formalin administration for 30 minutes. The time spent biting, licking, or lifting the injected hind paw was considered as nociceptive behavior and recorded (in seconds) during the first phase (0–5 min, neurogenic phase) and the second phase (15–30 min, inflammatory phase) of the test [24].

Prior to the administration of formalin (i.pl.), the animals were placed in a clear observation chamber for 20 minutes for adaptation. The extract was tested at 30, 60, and 120 mg/kg (p.o.). Diclofenac potassium (50 mg/kg, p.o.) was used as positive control [24]. The extract dosage that presented the best antinociceptive/anti-inflammatory activity in the formalin test was chosen to be used in the other behavioral tests.

### 2.5. Carrageenan-Induced Paw Oedema

This assay was performed according to Trevisan et al. [25]. In brief, the thickness of the right hind paw of each mouse was measured with a caliper prior to administration of the oral treatments. In this paw, 20 *μ*L of carrageenan (300 *μ*g/paw, diluted in saline) was injected 1 hour after the oral administration of the extract of *C. xanthocarpa* (60 mg/kg), indomethacin (positive control, 20 mg/kg), or vehicle [26].

The thickness (mm) of the paw oedema was assessed with a caliper at different time points (30 minutes, 1 hour, 2 hours, 4 hours, 6 hours, and 8 hours) after the intraplantar injection (i.pl.) of carrageenan and described as Δpaw thickness = test paw thickness–basal paw thickness [27].

### 2.6. Antichemostactic Assay *In Vitro*

Experiments were carried out according to the modified Boyden chamber method [28]. A total of seven animals were used in this assay. To obtain rat polymorphonuclear neutrophils, 10 mL of sterile 1% glycogen (w/v) was injected into the peritoneum of one Wistar rat that was euthanized for leukocytes collection 4 h later. Prior to the chemotaxis assay, neutrophils were treated with the extract (concentrations of 0.1 to 10 *μ*g/mL) and indomethacin (10 *μ*g/mL) at 37°C for 30 min. To obtain plasma, six rats were used. The plasma was incubated at 37°C for 30 min with 65 *μ*g/mL of LPS (lipopolysaccharide from *E. coli*) and diluted in Hanks buffer to a 20% solution (v/v). The leukocyte/samples were added in the upper wells of the chamber, separated by an 8 *μ*m nitrocellulose filter paper (Millipore, USA) from the chemotactic stimulant (LPS) present in the bottom compartment. Then, the chamber was kept at 37°C for 1 h. Migration of leucocytes through the filter was measured by using an optical microscope. The distance from the top of the filter to the farthest plane of focus containing two cells, in five microscopic fields of duplicate filters, allowed the evaluation of leukocyte migration (*µ*m).

The stock solution of the extract (1 mg/mL) was prepared by using Hanks' balanced salt solution (HBSS) with addition of 1% (v/v) Tween 80 and sonicated for 1 minute. The reference drug indomethacin was also dissolved in HBSS. The concentration of Tween 80 in all final working solutions was less than 0.01%. As negative control, the neutrophil solution was applied without addition of an antichemostatic agent as well as Tween 80 solution at the concentration used for sample dilution (1%).

### 2.7. Open Field Test

The open field test was performed in order to evaluate the possible effects of *C. xanthocarpa* seed extract on mice locomotor and exploratory activities, which could influence the results of the other behavioral tests. The apparatus used for the open field test was an acrylic box with the bottom divided into equal quadrants (40 × 30 × 30 cm). Mice were individually placed in the center of the apparatus and the number of crossings, rearings, groomings, and fecal bolus expelled from the animal during the observation time was registered [29]. Independent groups of mice were orally treated with the dose of the extract that presented the best anti-inflammatory results in the formalin and carrageenan tests (60 mg/kg), diclofenac potassium (50 mg/kg), or vehicle one hour before being evaluated in the open field.

### 2.8. Acute Toxicity

The assay was performed according to the OECD Guideline No. 423 [30]. The animals were orally treated with the extract of *C. xanthocarpa* at 2000 mg/kg (*n* = 6) or vehicle (*n* = 3). After the administration, the animals were monitored for 4 hours, with special attention to their somatomotor activity, alterations in skin, piloerection, eyes and mucous membranes, tremors, seizures, salivation, diarrhea, lethargy, sleep, and coma [30]. Mice were observed for 15 days after treatment, and their body weight and food intake were registered every 2 days. At the end of the experimental period, the animals were euthanized, and the macroscopic aspect of the organs (liver, kidneys, adrenal glands, spleen, lungs, heart, and brain) as well as their relative weights (%) was recorded.

### 2.9. Statistical Analysis

The results were analyzed by one-way analysis of variance (ANOVA) followed by the Student–Newman–Keuls test, except data from relative body weight and food intake, which were analyzed by two-way ANOVA with repeated measures. The evaluation of the relative weight of the organs was performed by unpaired *t*-test. GraphPad Prism 5.01 software was used for statistical analysis. Results are expressed as mean ± standard error of the mean (S.E.M.). Values of *p* < 0.05 were considered significant.

## 3. Results and Discussion

The supercritical CO_2_ (SCCO_2_) extraction is a promising, alternative method to conventional solvent extraction techniques due to its well-known benefits [31]. It is a process free of toxic waste, which does not cause thermal degradation of the extracts and does not require large energy costs as is the case in extraction processes with common solvents that require solvent evaporation (distillation) to obtain the final extract [32, 33].

In addition, the increased extraction efficiency by the influence of pressure and/or temperature on the solubility of several compounds is related to SCCO_2_ extraction, which may be more selective when these parameters are optimized according to the plant used [32, 33]. It is also important to note that SCCO_2_ extraction favors a solvent-free product since CO_2_ is removed during the depressurizing process, generating no residues, being a clean and alternative method [34]. The operating conditions of the extraction performed by Capelleto et al. [18] (250 bar, density of 0.879 kg/m^3^) give the fluid a solvating power similar to a conventional solvent like hexane, allowing the extraction of nonpolar chemical compounds such as terpenes [35]. The extraction yield using supercritical CO_2_ was 8.02 ± 0.05 wt%, and the major components of *C. xanthocarpa* extract obtained by SCCO_2_ were *β*-caryophyllene (11.67%), followed by *γ*-cadinene (9.58%), *α*-cadinol (7.17%), viridiflorol (6.70%), and *δ*-gurjunene (6.48%), as previously reported by Capelleto et al. [18].

Considering that seeds are discarded frequently, once they are a byproduct of the industry, the use of *C. xanthocarpa* seeds in the extraction could represent an alternative to reduce the environmental waste, being economical and safe for the environment [36]. A relevant note is that the seeds' compounds could be used by the pharmaceutical and food industries, which emphasizes the importance of improving the technological extraction and characterization of its chemical composition [37]. In agreement with this observation, the SCCO_2_ extract of *C. xanthocarpa* seeds demonstrated a promising pharmacological activity in the present study.

**Figure 1:**
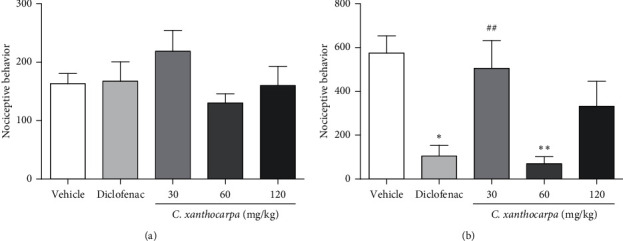
Effect of *Campomanesia xanthocarpa* seed extract obtained by supercritical CO_2_ in mice formalin test. Nociceptive behavior was considered as the time (s) of elevation, biting, or licking of the paw in the first phase ((a), 0–5 min) or second phase ((b), 15–30 min) of the test. The animals (*n* = 4–7/group) were orally treated with vehicle (0.9% NaCl + 1% Tween 80), diclofenac potassium (50 mg/kg), or *C. xanthocarpa* seed extract (30, 60, or 120 mg/kg) 1 hour prior to the intraplantar administration of formalin 2%. One-way ANOVA, *post hoc* Student–Newman–Keuls: ^*∗*^*p* < 0.05 and ^*∗∗*^*p* < 0.01 compared to the vehicle-treated group and ^##^*p* < 0.01 compared to the diclofenac-treated group. Results are expressed as mean ± SEM.

The first phase of formalin test is related to neurogenic pain, elicited by the activation of nociceptive fibers. At this phase, none of the tested doses of *C. xanthocarpa* extract presented a significant antinociceptive effect (Figure 1(a)). The second phase of the test is associated with the action of inflammatory mediators (prostaglandin, histamine, and bradykinin) [38]. In that phase, the extract-treated group (*p* < 0.01) as well as the group that received diclofenac potassium (*p* < 0.05) presented a significant reduction in the time of nociceptive behavior in comparison with the vehicle-treated animals (Figure 1(b)).

Considering the extract was effective on the reduction of nociceptive behavior induced by formalin during the second (inflammatory) phase of the formalin test, we suggest that its effectiveness is better in alleviating the pain elicited by inflammation. This anti-inflammatory effect was presented as a U-shaped curve in the test, and once the animals are treated with the extract at 30 and 120 mg/kg, they did not present significant decrease in nociceptive behavior (Figure 1(b)). Evidence shows that the dose response for pain-related endpoints is frequently biphasic, being independent of the animal model used, endpoint measured, or antinociceptive substance tested. This phenomenon is usually observed with substances that activate cannabinoid receptors, such as cannabidiol (CB1 agonist) [39–41]. Considering that *β*-caryophyllene (CB2 agonist) is the major compound found in *C. xanthocarpa* seed extract, we may infer that this molecule contributes to the U-shaped curve found in our study. This hypothesis is corroborated by the findings of Klauke et al. [20], which demonstrated that a low dose of *β*-caryophyllene was more effective than higher doses in a thermal hyperalgesia mouse model. The mechanisms that might explain this fact are still not completely elucidated, but include dose-dependent changes in receptor occupation, resulting in differential activation of intracellular signaling cascades and thus in distinct physiological outcomes [41, 42].

**Figure 2:**
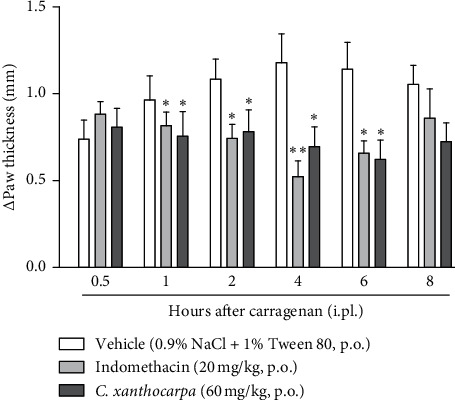
Effect of *Campomanesia xanthocarpa* seed extract obtained by supercritical CO_2_ on carrageenan-induced paw oedema in mice. The animals (*n* = 10–14/group) were treated with vehicle (0.9% NaCl + 1% Tween 80, 10 mL/kg), indomethacin (20 mg/kg), or *C. xanthocarpa* seed extract (60 mg/kg) 1 hour before carrageenan (300 *μ*g/paw) intraplantar administration. Paw thickness (mm) was measured 30 minutes, 1 hour, 2 hours, 4 hours, 6 hours, and 8 hours after carrageenan injection. One-way ANOVA, *post hoc* Student–Newman–Keuls: ^*∗*^*p* < 0.05 and ^*∗∗*^*p* < 0.01 compared to the vehicle-treated group. Results are expressed as mean ± SEM.

Additionally, we performed the carrageenan-induced paw oedema test (Figure 2) with the extract at 60 mg/kg, which was the only dose that presented a significant anti-inflammatory action in the formalin test (phase 2, Figure 1(b)). One hour after the administration of carrageenan, there was a significant reduction in paw oedema in the indomethacin and *C. xanthocarpa* extract-treated groups when compared to the group that received vehicle (*p* < 0.05). This difference remained significant up to 6 hours after administration (Figure 2).

In the carrageenan test, two phases can be detected: the first one is related to carrageenan-induced oedematogenic response, which results from the rapid production of several inflammatory mediators, such as histamine, serotonin, and bradykinin. In the second phase (after 3 hours of the injection), there is a significant release of prostaglandins and nitric oxide (NO), produced by inducible isoforms of COX (COX-2) and nitric oxide synthase (iNOS), respectively [43]. Considering that the effects of *C. xanthocarpa* seed extract were more pronounced in the second phase of the carrageenan test, these results suggest that the action of *C. xanthocarpa* may be related to the inhibition of prostaglandin release or to the nitric oxide production. These results are in agreement with those from the formalin test, where the extract was effective only in the second phase of the assay, which is related to inflammatory pain [21].

**Figure 3:**
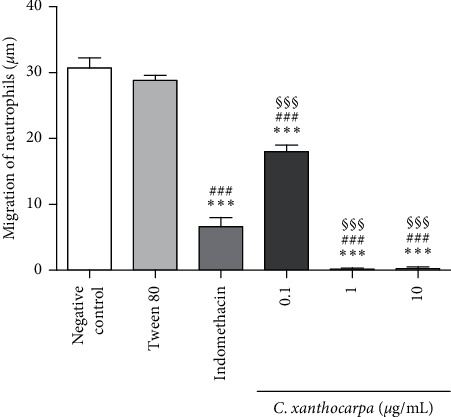
Effects of *Campomanesia xanthocarpa* seed extract obtained by supercritical CO_2_ on the polymorphonuclear neutrophil chemotaxis *in vitro*. The leukocytes were treated with a range of 0.1–1.0 *µ*g/mL of extract at 37°C for 1 h. Chemotaxis is expressed as mean ± SEM of neutrophils migration (µm). One-way ANOVA *post hoc* Student–Newman–Keuls: ^*∗∗∗*^*p* < 0.001 compared to the negative control; ^###^*p* < 0.001 compared to Tween 80; and ^$$$^*p* < 0.001 compared to indomethacin (10 *μ*g/mL, positive control).

The chemotaxis assay is performed to assess substances' ability to inhibit the migration of cells involved in inflammation [28]. The results obtained in the Boyden chamber test are shown in Figure 3.

The *C. xanthocarpa* seed extract showed significant inhibition of neutrophil migration relative to the negative control (*p* < 0.05) at all concentrations tested. The extract presented an inhibitory activity of 100% at 1 and 10 *µ*g/mL and 41.6% at 0.1 *µ*g/mL. In addition, at 1 and 10 *µ*g/mL, the extract showed antichemotactic activity in significantly (*p* < 0.001) higher percentages than the positive control, indomethacin (inhibition of cell migration of 78.4%). The leukocytes migration to the site of injury is considered one of the major stages implicated in the inflammation process, and these cells are involved in the early stages of inflammation [44]. In this sense, our results indicate that the *C. xanthocarpa* seed extract acts in the acute phase of inflammatory process, by inhibiting neutrophil chemotaxis.

*β*-Caryophyllene, the main constituent of *C. xanthocarpa* seed extract, presents several biological activities, such as anti-inflammatory [45], bactericidal [46], and antitumoral [47] activities. According to Klauke et al. [20], the mechanism of anti-inflammatory action of *β*-caryophyllene is related to its selective agonist action of CB2 cannabinoid receptors that are predominantly expressed in immune cells. It is known that the activation of CB2 receptors from immune cells inhibits the release of inflammatory mediators, resulting in marked analgesia [48], reduction of leukocytes chemotaxis, and oedema formation [49]. Furthermore, the selective activation of CB2 receptors elicits anti-inflammatory effects with no neurobehavioral side effects related to psychotomimetic cannabinoid action and shows antinociceptive effects in several pain animal models [50]. Also, it is evident that *β*-caryophyllene can modulate cell migration, the production of proinflammatory mediators and the activation of intracellular signaling pathways. It has been shown that *β*-caryophyllene mainly inhibits the influx of neutrophils to the inflammatory site, in experimental models of colitis, paw oedema, and renal inflammation [51, 52]. Moreover, the anti-inflammatory action of *β*-caryophyllene can also be attributed to cyclooxygenase enzyme inhibitory properties and decreased production of prostaglandins [20]. Considering these findings, we may infer that *β*-caryophyllene contributes to the *in vitro* antichemotactic effects of the extract, as well as to its *in vivo* anti-inflammatory effects.

Considering the chemical compounds of the *C. xanthocarpa* seed extract obtained by SCCO_2_, it is possible to infer that other constituents could also be related to the anti-inflammatory action of the seeds, such as viridiflorol, spathulenol, and linalool. Trevizan et al. [53] demonstrated that the essential oil of *Allophylus edulis* leaves, enriched in viridiflorol, has antimicrobial, antioxidant, and anti-inflammatory activities in mice. Moreover, Do Nascimento et al. [54] showed that the essential oil of *Psidium guineense* Sw. and spathulenol, the main component of the oil, presents antioxidant, antiproliferative, and anti-inflammatory properties in mice. Likewise, linalool exhibits anti-inflammatory activity in rats [55].

In addition to the results found in the present study, Capeletto et al. [18] demonstrated that the extract of *C. xanthocarpa* seeds obtained by SCCO_2_ shows antioxidant and antimicrobial action, which may be related to its chemical constituents, mainly terpenoids, such as *β*-caryophyllene. Regarding the other chemical compounds present in the extract, such as *δ*-gurjunene, aromadendrene, *γ*-cadinene, and *δ*-cadinene, there are no reports about anti-inflammatory activity that could be attributed to them.

**Figure 4:**
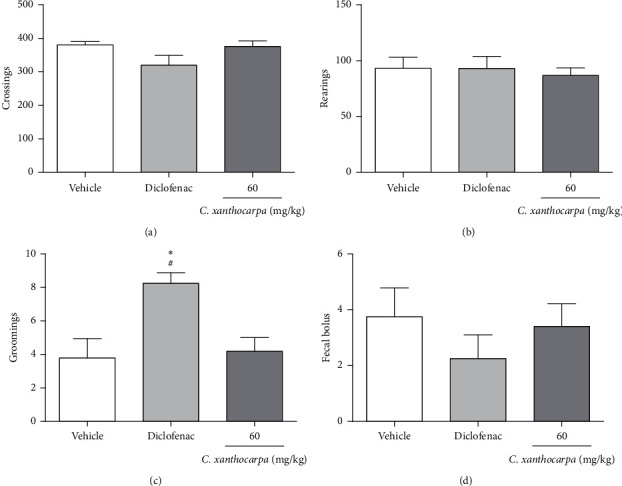
Effect of *Campomanesia xanthocarpa* seed extract obtained by supercritical CO_2_ on the locomotor activity (open field test) of mice: (a) number of crossings, (b) number of rearings, (c) number of groomings, and (d) number of fecal bolus at the end of the test. The mice (*n* = 5/group) were orally treated with vehicle (0.9% NaCl + 1% Tween 80, 10 mL/kg), diclofenac potassium (50 mg/kg), or extract (60 mg/kg) 1 h before the test. One-way ANOVA followed by Student–Newman–Keuls test: ^*∗*^*p* < 0.05 compared to the vehicle-treated group and ^#^*p* < 0.05 compared to the group treated with the extract. Results are expressed as mean ± SEM.

The possible effects of the *C. xanthocarpa* seed extract on mice locomotor and exploratory activities was assessed in the open field test. The animals received (p.o.) the extract at 60 mg/kg, the dose that presented a significant anti-inflammatory action. There were no differences in the number of crossings (Figure 4(a)), rearings (Figure 4(b)), groomings (Figure 4(c)), and fecal bolus (Figure 4(d)) expelled by the extract-treated animals when compared to the groups that were treated with vehicle. These findings demonstrate that the extract does not induce motor alterations that could interfere in the assessment of nociception.

**Figure 5:**
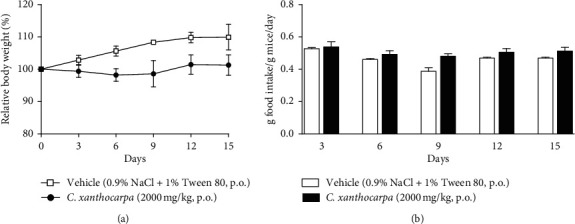
Effect of *Campomanesia xanthocarpa* seed extract obtained by supercritical CO_2_ acute treatment (2000 mg/kg, p.o.) on female mice food intake (a) (g food intake/g mice/day) and relative body weight (%) (b). Data are expressed as mean ± S.E.M. (*n* = 3–6 mice/group). Two-way repeated measures ANOVA *post hoc* Student–Newman–Keuls: ^*∗∗*^*p* < 0.01 different from the first measure (day 0) in the same group of treatment.

The effect of the extract on the relative body weight (%) and food intake assessed in the acute toxicity test is shown in Figures 5(a) and 5(b), respectively. Mice treated with vehicle presented a significant (*p* < 0.01) increase in the body weight (Figure 4(a)) at the 9^th^, 12^th^, and 15^th^ days of observation in comparison with the initial weight. There were no changes in body weight of the extract-treated animals during the period of observation. Significant decreases or increases in body weight might be related to toxicity of substances [56], but the extract did not trigger any significant change in the body weight of mice. Moreover, no differences in the food intake were detected over the observation period between the group treated with *C. xanthocarpa* extract and the group treated with vehicle (Figure 5(b)). Also, no death was recorded.

**Figure 6:**
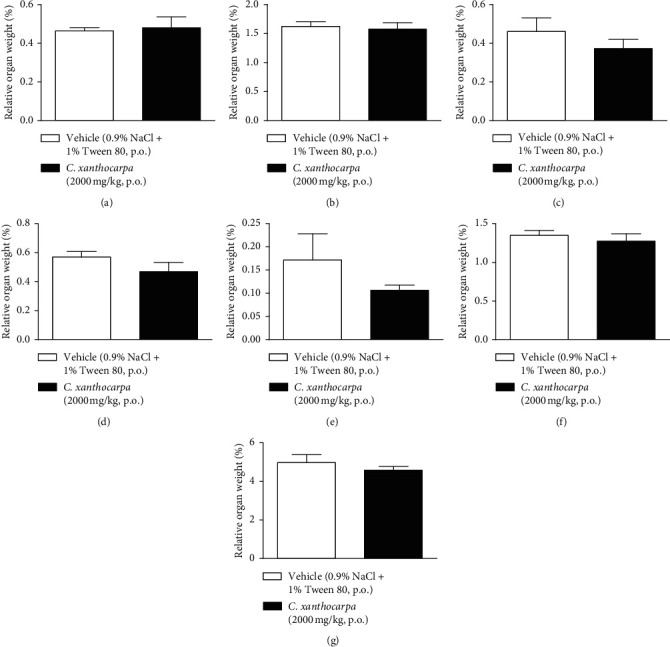
Effect of *Campomanesia xanthocarpa* seed extract obtained by supercritical CO_2_ on the relative (%) organs' weight of female Swiss mice in the acute oral toxicity test. Mice (*n* = 3–6) were treated with vehicle (0.9% NaCl + 1% Tween 80, 10 mL/kg) and *C. xanthocarpa* seed extract (2000 mg/kg). The relative weight of heart (a), brain (b), thymus (c), spleen (d), adrenals (e), kidneys (f), and liver (g) was evaluated 15 days after treatment. Unpaired *t*-test. Results are expressed as mean ± SEM.

The results of the relative weight (%) of the organs of the animals treated with *C. xanthocarpa* or vehicle are shown in Figure 6. There were no significant macroscopic changes and no differences in the relative weight of the heart (Figure 6(a)), brain (Figure 6(b)), thymus (Figure 6(c)), spleen (Figure 6(d)), adrenal glands (Figure 6(e)), and liver (Figure 6(g)) between the vehicle-treated group and the group treated with *C. xanthocarpa* extract (2000 mg/kg).

Da Silva et al. [17] demonstrated that a hydroalcoholic extract from the leaves of *C. xanthocarpa* is devoid of acute toxicity in mice. The extract did not cause behavioral alterations nor changes in the food consumption and water uptake. Moreover, no significant differences in the absolute and relative weight of organs (heart, lung, spleen, liver, and kidneys) were found in the animals that received the *C. xanthocarpa* leaves hydroalcoholic extract. These results are in agreement with those obtained in our study. Considering that no gross alterations in the animals' behavior and no deaths were recorded during the observation period after treatment, the extract of *C. xanthocarpa* leaves obtained by SCCO_2_ can be classified according to OECD Guideline No. 423 (2001) [30] in category 5 (LD_50_ is between 2000 and 5000 mg/kg).

## 4. Conclusions

Taken together, our results demonstrate that *Campomanesia xanthocarpa* seed extract obtained by supercritical CO_2_ presents a promising anti-inflammatory activity. In addition, the *C. xanthocarpa* seed extract is devoid of acute toxicity in mice. Lastly, the seeds of *C. xanthocarpa* fruit could represent a source of anti-inflammatory compounds.

## Data Availability

The data used to support the findings of this study are available from the corresponding author upon request.

## References

[1] Lima D. K. S., Ballico L. J., Rocha Lapa F. (2012). Evaluation of the antinociceptive, anti-inflammatory and gastric antiulcer activities of the essential oil from *Piper aleyreanum* C.DC in rodents. *Journal of Ethnopharmacology*.

[2] World Health Organization (2014). *C-reactive Protein Concentrations as a Marker of Inflammation or Infection for Interpreting Biomarkers of Micronutrient Status*.

[3] Marmitt D. J., Rempel C., Goettert M. I., Silva A. C. (2015). *Plantas Medicinais da RENISUS Com Potencial Anti-inflamatório: Revisão Sistemática Em Três Bases de Dados Científicas*.

[4] Singh G. (2000). Gastrointestinal complications of prescription and over-the-counter nonsteroidal anti-inflammatory drugs. *American Journal of Therapeutics*.

[5] Hunt R., B Lazebnik L., C Marakhouski Y. (2018). International consensus on guiding recommendations for management of patients with nonsteroidal antiinflammatory drugs induced gastropathy-ICON-G. *Euroasian Journal of Hepato-Gastroenterology*.

[6] Barnett R. (2016). Cushing’s syndrome. *The Lancet*.

[7] Maroon J. C., Bost J. W., Maroon A. (2010). Natural anti-inflammatory agents for pain relief. *Surgical Neurology International*.

[8] Rodrigues T., Reker D., Schneider P., Schneider G. (2016). Counting on natural products for drug design. *Nature Chemistry*.

[9] Biavatti M. W., Farias C., Curtius F. (2004). Preliminary studies on *Campomanesia xanthocarpa* (Berg.) and *Cuphea carthagenensis* (Jacq.) J.F. Macbr. aqueous extract: weight control and biochemical parameters. *Journal of Ethnopharmacology*.

[10] Dickel M. L., Rates S. M. K., Ritter M. R. (2007). Plants popularly used for loosing weight purposes in Porto Alegre, South Brazil. *Journal of Ethnopharmacology*.

[11] Trojan-Rodrigues M., Alves T. L. S., Soares G. L. G., Ritter M. R. (2012). Plants used as antidiabetics in popular medicine in Rio Grande do Sul, southern Brazil. *Journal of Ethnopharmacology*.

[12] Souza-Moreira T. M., Salvagnini L. E., Santos E. (2011). Antidiarrheal activity of Campomanesia xanthocarpa fruit. *Journal of Medicinal Food*.

[13] Silva B. E. O., Bacchi E. M., Kato E. T. M. (2004). Antiulcerogenic effects of *Campomanesia xanthocarpa*. *Journal of Ethnopharmacology*.

[14] Otero J. S., Hirsch G. E., Klafke J. Z. (2017). Inhibitory effect of *Campomanesia xanthocarpa* in platelet aggregation: comparison and synergism with acetylsalicylic acid. *Thrombosis Research*.

[15] Viecili P. R. N., Borges D. O., Kirsten K. (2014). Effects of *Campomanesia xanthocarpa* on inflammatory processes, oxidative stress, endothelial dysfunction and lipid biomarkers in hypercholesterolemic individuals. *Atherosclerosis*.

[16] Salmazzo G. R., Verdan M. H., Silva F. (2019). Chemical composition and antiproliferative, antioxidant and trypanocidal activities of the fruits from Campomanesia xanthocarpa (Mart.) O. Berg (Myrtaceae). *Natural Product Research*.

[17] da Silva É. R. S., Salmazzo G. R., da Silva Arrigo J., Oliveira R. J., Kassuya C. A. L., Cardoso C. A. L. (2016). Anti-inflammatory evaluation and toxicological analysis of Campomanesia xanthocarpa Berg. *Inflammation*.

[18] Capeletto C., Conterato G., Scapinello J. (2016). Chemical composition, antioxidant and antimicrobial activity of guavirova (*Campomanesia xanthocarpa* Berg) seed extracts obtained by supercritical CO2 and compressed n-butane. *The Journal of Supercritical Fluids*.

[19] Scapinello J., Oliveira J. V., Ribeiros M. L., Tomazelli O., Chiaradia L. A., Dal Magro J. (2014). Effects of supercritical CO_2_ extracts of Melia azedarach L. on the control of fall armyworm (Spodoptera frugiperda). *The Journal of Supercritical Fluids*.

[20] Klauke A.-L., Racz I., Pradier B. (2014). The cannabinoid CB2 receptor-selective phytocannabinoid beta-caryophyllene exerts analgesic effects in mouse models of inflammatory and neuropathic pain. *European Neuropsychopharmacology*.

[21] Hunskaar S., Hole K. (1987). The formalin test in mice: dissociation between inflammatory and non-inflammatory pain. *Pain*.

[22] Tjølsen A., Berge O. G., Hunskaar S., Rosland J. H., Hole K. (1992). The formalin test: an evaluation of the method. *Pain*.

[23] Santos A. R. S., Calixto J. B. (1997). Further evidence for the involvement of tachykinin receptor subtypes in formalin and capsaicin models of pain in mice. *Neuropeptides*.

[24] Izquierdo T., Espinosa de los Monteros-Zuñiga A., Cervantes-Durán C., Lozada M. C., Godínez-Chaparro B. (2013). Mechanisms underlying the antinociceptive effect of mangiferin in the formalin test. *European Journal of Pharmacology*.

[25] Trevisan G., Rossato M. F., Walker C. I. B. (2012). Identification of the plant steroid *α*-spinasterol as a novel transient receptor potential vanilloid 1 antagonist with antinociceptive properties. *Journal of Pharmacology and Experimental Therapeutics*.

[26] Batista E. K. F., Trindade H. I., Lira S. R. S., Muller J. B. B. S., Silva L. L. B., Batista M. C. S. (2016). Atividades antinociceptiva e antiinflamatória do extrato etanólico de Luehea divaricata. *Revista Brasileira de Plantas Medicinais*.

[27] Scapinello J., Müller L. G., Schindler M. S. Z. (2019). Antinociceptive and anti-inflammatory activities of philodendron bipinnatifidum schott ex endl (araceae). *Journal of Ethnopharmacology*.

[28] Suyenaga E., Konrath E., Dresch R. (2011). Appraisal of the antichemotactic activity of flavonoids on polymorphonuclear neutrophils. *Planta Medica*.

[29] Müller L. G., Salles L. A., Stein A. C. (2012). Antidepressant-like effect of valeriana glechomifolia meyer (valerianaceae) in mice. *Progress in Neuro-Psychopharmacology and Biological Psychiatry*.

[30] OECD (2001). *OECD Guideline for Testing of Chemicals. Acute Oral Toxicity—Acute Toxic Class Method*.

[31] Liu J., Lin S., Wang Z. (2011). Supercritical fluid extraction of flavonoids from Maydis stigma and its nitrite-scavenging ability. *Food and Bioproducts Processing*.

[32] Koubaa M., Mhemdi H., Fages J. (2018). Recovery of valuable components and inactivating microorganisms in the agro-food industry with ultrasound-assisted supercritical fluid technology. *The Journal of Supercritical Fluids*.

[33] Gandhi K., Arora S., Kumar A. N. M. (2017). Industrial applications of supercritical fluid extraction: a review. *International Journal of Chemical Studies*.

[34] Farías-Campomanes A. M., Rostagno M. A., Coaquira-Quispe J. J., Meireles M. A. A. (2015). Supercritical fluid extraction of polyphenols from lees: overall extraction curve, kinetic data and composition of the extracts. *Bioresources and Bioprocessing*.

[35] Lazarotto M., Valério A., Boligon A. (2018). Chemical composition and antibacterial activity of bergamot peel oil from supercritical CO2 and compressed propane extraction. *The Open Food Science Journal*.

[36] Schieber A., Stintzing F. C., Carle R. (2001). By-products of plant food processing as a source of functional compounds - recent developments. *Trends in Food Science & Technology*.

[37] Kobori C. N., Jorge N. (2005). Caracterização dos óleos de algumas sementes de frutas como aproveitamento de resíduos industriais. *Ciência e Agrotecnologia*.

[38] de Mesquita Padilha M., Vilela F. C., da Silva M. J. D., dos Santos M. H., Alves-da-Silva G., Giusti-Paiva A. (2009). Antinociceptive effect of the extract of *Morus nigra* leaves in mice. *Journal of Medicinal Food*.

[39] Calabrese E. J. (2008). Pain and U-shaped dose responses: occurrence, mechanisms, and clinical implications. *Critical Reviews in Toxicology*.

[40] Malfait A. M., Gallily R., Sumariwalla P. F. (2000). The nonpsychoactive cannabis constituent cannabidiol is an oral anti-arthritic therapeutic in murine collagen-induced arthritis. *Proceedings of the National Academy of Sciences*.

[41] Sulcova E., Mechoulam R., Fride E. (1998). Biphasic effects of anandamide. *Pharmacology Biochemistry and Behavior*.

[42] Beltramo M., Bernardini N., Bertorelli R. (2006). CB2 receptor-mediated antihyperalgesia: possible direct involvement of neural mechanisms. *European Journal of Neuroscience*.

[43] Seibert K., Zhang Y., Leahy K. (1994). Pharmacological and biochemical demonstration of the role of cyclooxygenase 2 in inflammation and pain. *Proceedings of the National Academy of Sciences*.

[44] Boudiaf K., Hurtado-Nedelec M., Belambri S. A. (2016). Thymoquinone strongly inhibits fMLF-induced neutrophil functions and exhibits anti-inflammatory properties *in vivo*. *Biochemical Pharmacology*.

[45] Oliveira-Tintino C. D. d. M., Pessoa R. T., Fernandes M. N. M. (2018). Anti-inflammatory and anti-edematogenic action of the Croton campestris A. St.-Hil (Euphorbiaceae) essential oil and the compound *β*-caryophyllene in in vivo models. *Phytomedicine*.

[46] Kang R., Helms R., Stout M. J., Jaber H., Chen Z., Nakatsu T. (1992). Antimicrobial activity of the volatile constituents of Perilla frutescens and its synergistic effects with polygodial. *Journal of Agricultural and Food Chemistry*.

[47] Zheng G.-Q., Kenney P. M., Lam L. K. T. (1992). Sesquiterpenes from clove (eugenia caryophyllata) as potential anticarcinogenic agents. *Journal of Natural Products*.

[48] Ibrahim M. M., Porreca F., Lai J. (2005). CB2 cannabinoid receptor activation produces antinociception by stimulating peripheral release of endogenous opioids. *Proceedings of the National Academy of Sciences*.

[49] Iwamura H., Suzuki H., Ueda Y., Kaya T., Inaba T. (2001). *In vitro* and *in vivo* pharmacological characterization of JTE-907, a novel selective ligand for cannabinoid CB2 receptor. *Journal of Pharmacology and Experimental Therapeutics*.

[50] Maurya N., Velmurugan B. K. (2018). Therapeutic applications of cannabinoids. *Chemico-biological Interactions*.

[51] Bento A. F., Marcon R., Dutra R. C. (2011). *β*-Caryophyllene inhibits dextran sulfate sodium-induced colitis in mice through CB2 receptor activation and PPAR*γ* pathway. *The American Journal of Pathology*.

[52] Horváth B., Mukhopadhyay P., Kechrid M. (2012). *β*-Caryophyllene ameliorates cisplatin-induced nephrotoxicity in a cannabinoid 2 receptor-dependent manner. *Free Radical Biology and Medicine*.

[53] Trevizan L. N. F., Nascimento K. F. d., Santos J. A. (2016). Anti-inflammatory, antioxidant and anti- Mycobacterium tuberculosis activity of viridiflorol: the major constituent of Allophylus edulis (A. St.-Hil., A. Juss. & Cambess.) Radlk. *Journal of Ethnopharmacology*.

[54] do Nascimento K. F., Moreira F. M. F., Alencar Santos J. (2018). Antioxidant, anti-inflammatory, antiproliferative and antimycobacterial activities of the essential oil of Psidium guineense Sw. and spathulenol. *Journal of Ethnopharmacology*.

[55] Peana A. T., D’Aquila P. S., Panin F., Serra G., Pippia P., Moretti M. D. L. (2002). Anti-inflammatory activity of linalool and linalyl acetate constituents of essential oils. *Phytomedicine*.

[56] Kifayatullah M., Mustafa M. S., Sengupta P., Sarker M. M. R., Das A., Das S. K. (2015). Evaluation of the acute and sub-acute toxicity of the ethanolic extract of Pericampylus glaucus (Lam.) Merr. in BALB/c mice. *Journal of Acute Disease*.

